# Guided Web-Based Cognitive Behavior Therapy for Perfectionism: Results From Two Different Randomized Controlled Trials

**DOI:** 10.2196/jmir.9823

**Published:** 2018-04-26

**Authors:** Alexander Rozental, Roz Shafran, Tracey D Wade, Radha Kothari, Sarah J Egan, Linda Ekberg, Maria Wiss, Per Carlbring, Gerhard Andersson

**Affiliations:** ^1^ Department of Psychology Stockholm University Stockholm Sweden; ^2^ Institute of Child Health University College London London United Kingdom; ^3^ School of Psychology Flinders University Adelaide Australia; ^4^ School of Psychology Curtin University Perth Australia; ^5^ Department of Behavioural Sciences and Learning Linköping University Linköping Sweden; ^6^ Department of Clinical Neuroscience Karolinska Institutet Stockholm Sweden

**Keywords:** cognitive behavior therapy, internet, perfectionism, follow-up studies, cognitive therapy

## Abstract

**Background:**

Perfectionism can become a debilitating condition that may negatively affect functioning in multiple areas, including mental health. Prior research has indicated that internet-based cognitive behavioral therapy can be beneficial, but few studies have included follow-up data.

**Objective:**

The objective of this study was to explore the outcomes at follow-up of internet-based cognitive behavioral therapy with guided self-help, delivered as 2 separate randomized controlled trials conducted in Sweden and the United Kingdom.

**Methods:**

In total, 120 participants randomly assigned to internet-based cognitive behavioral therapy were included in both intention-to-treat and completer analyses: 78 in the Swedish trial and 62 in the UK trial. The primary outcome measure was the Frost Multidimensional Perfectionism Scale, Concern over Mistakes subscale (FMPS CM). Secondary outcome measures varied between the trials and consisted of the Clinical Perfectionism Questionnaire (CPQ; both trials), the 9-item Patient Health Questionnaire (PHQ-9; Swedish trial), the 7-item Generalized Anxiety Disorder scale (GAD-7; Swedish trial), and the 21-item Depression Anxiety Stress Scale (DASS-21; UK trial). Follow-up occurred after 6 months for the UK trial and after 12 months for the Swedish trial.

**Results:**

Analysis of covariance revealed a significant difference between pretreatment and follow-up in both studies. Intention-to-treat within-group Cohen *d* effect sizes were 1.21 (Swedish trial; 95% CI 0.86-1.54) and 1.24 (UK trial; 95% CI 0.85-1.62) for the FMPS CM. Furthermore, 29 (59%; Swedish trial) and 15 (43%; UK trial) of the participants met the criteria for recovery on the FMPS CM. Improvements were also significant for the CPQ, with effect sizes of 1.32 (Swedish trial; 95% CI 0.97-1.66) and 1.49 (UK trial; 95% CI 1.09-1.88); the PHQ-9, effect size 0.60 (95% CI 0.28-0.92); the GAD-7, effect size 0.67 (95% CI 0.34-0.99); and the DASS-21, effect size 0.50 (95% CI 0.13-0.85).

**Conclusions:**

The results are promising for the use of internet-based cognitive behavioral therapy as a way of targeting perfectionism, but the findings need to be replicated and include a comparison condition.

## Introduction

Perfectionism has many positive features, such as striving for excellence, but it can also have a negative impact in many areas, including mental health [1]. Characterized by perfectionistic strivings and perfectionistic concerns, perfectionism, instead of helping the individual fulfill their goals, is associated with avoidance, worry, procrastination, and self-criticism [2]. Perfectionism has also been found to be related to anxiety disorders, depression, and eating disorders, in part by increasing the odds of their occurrence, but also by interfering with treatment progress, particularly by making behavior change more difficult. In particular, certain populations tend to have elevated perfectionism, such as people with eating disorders, depression, social anxiety disorder, panic disorder, and obsessive-compulsive disorder, compared with healthy controls [3]. It has therefore been suggested that the use of psychological treatments to address perfectionism could improve outcomes for other conditions as well through transdiagnostic processes. Moreover, the two higher-order dimensions of perfectionism have also been found to be related to psychiatric disorders in different ways. Perfectionistic strivings seem to be particularly related to eating disorders, while perfectionism concerns are primarily associated with depression and anxiety disorders [2]. This could imply that psychological treatments targeting perfectionism might have to be adapted depending on the psychiatric disorder [3]. Furthermore, elevated levels of perfectionism can also be found among athletes and specific sociodemographics [2], suggesting that there may be populations that are particularly vulnerable to developing problems due to perfectionism, and for whom psychological intervention could prevent the development of further psychopathology.

A systematic review and meta-analysis by Lloyd et al [4] provided evidence for the efficacy of cognitive behavioral therapy (CBT) for managing perfectionism and for targeting symptoms of depression and anxiety. Data gathered from 6 clinical trials that used the Frost Multidimensional Perfectionism Scale, Concern over Mistakes subscale (FMPS CM) [5], a widely distributed self-report measure of perfectionism, showed that the average within-group Hedges *g* effect size between pre- and posttreatment was 1.32 (95% CI 1.02-1.64). Using the Clinical Perfectionism Questionnaire (CPQ), a self-report measure to assess clinical perfectionism [6], 3 clinical trials obtained comparable outcomes, with Hedges *g* effect sizes ranging from 0.90 to 1.24. However, even though the findings are promising, the meta-analysis did not provide any estimates for follow-up data, making it unclear whether the outcomes were maintained. A clinical trial by Egan et al [7], which recruited participants through self-referral and included an assessment at 6 months following treatment, indicated that CBT, administered face-to-face or via the internet without any guidance, had within-group Cohen *d* effect sizes of 2.11 (face-to-face; 95% CI 1.26-2.88) and 0.43 (unguided self-help; 95% CI –0.28 to 1.12) on the FMPS CM. For the CPQ, Cohen *d* effect sizes were 1.61 (face-to-face; 95% CI 0.83-2.32) and 0.73 (unguided self-help; 95% CI –0.01 to 1.42). In terms of recovery, based on a statistical cutoff and exceeding the Reliable Change Index (RCI), 67% (face-to-face) and 40% (unguided self-help) met the criteria for recovery at follow-up. This implies that the benefits can be maintained and possibly even improved from posttreatment. However, given the small sample size and high attrition rate, these findings need to be interpreted cautiously.

To extend the understanding of the treatment of perfectionism and to evaluate the efficacy of internet-based CBT (ICBT), 2 clinical trials of treatment with guided self-help were conducted in Sweden and the United Kingdom. The participants were self-referred and assigned to self-help with guidance from a therapist or to a waitlist control group. The results from pre- to posttreatment were encouraging, obtaining within-group Cohen *d* effect sizes of 1.03 (Swedish trial; 95% CI 0.69-1.36) and 1.47 (UK trial; 95% CI 1.06-1.86) for the FMPS CM. For the CPQ, Cohen *d* effect sizes were 1.44 (Swedish trial; 95% 1.08-1.78) and 1.67 (UK trial; 95% CI 1.25-2.07) [8,9].

This study aimed to assess the long-term benefits of treating perfectionism by determining the outcomes at follow-up for those who received ICBT with guided self-help in the Swedish and UK studies. However, given the differences between the 2 clinical trials on several key characteristics, for example their inclusion and exclusion criteria, we present the results separately rather than combined.

## Methods

### Participants and Procedure

Participants in both clinical trials consisted of self-referrals recruited through advertisements in the media, social media, and on campus grounds. Those interested in participating entered a website to complete a screening process and provide electronic informed consent. The websites were connected to a secure Web-based interface where self-report measures were presented, asynchronous communication with the study supervisors was carried out, and treatment content was delivered [10]. To log on, the participants had to use an autogenerated identification code (eg, 1234abcd), a strong personal password, and a one-time personal identification number sent to their mobile phone, ensuring safety and anonymity. Inclusion and exclusion criteria differed somewhat between the clinical trials, as did a few other key characteristics (see Table 1) [11,12].

**Figure 1:**
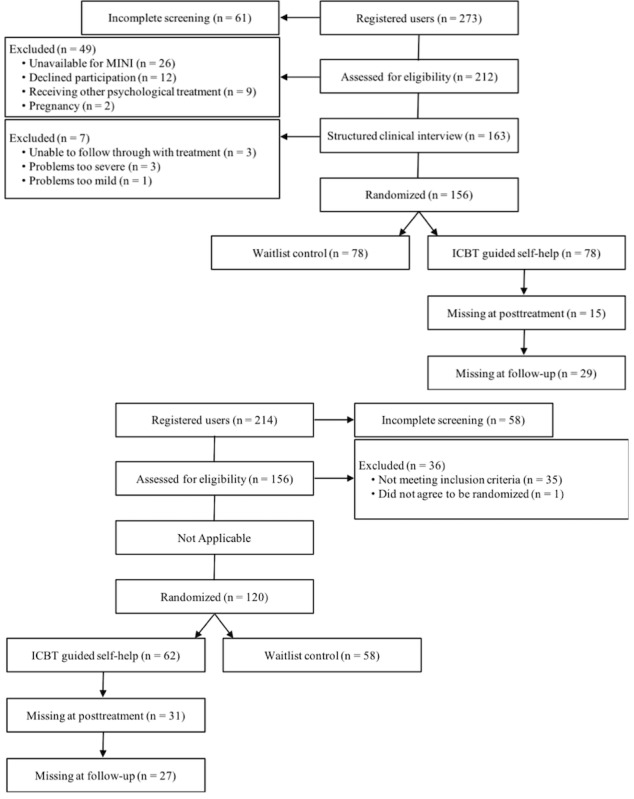
Flow of participants through the study. ICBT: Internet-based cognitive behavioral therapy; MINI: Mini-International Neuropsychiatric Interview.

Individuals fulfilling the inclusion criteria and being deemed eligible to participate were randomly assigned to ICBT with guided self-help or to a waitlist control group. In total, 156 were included in the Swedish trial (guided self-help n=78), compared with 120 in the UK trial (guided self-help n=62). Those assigned to the waitlist control later received the same treatment content, either in a second wave of treatment (Swedish trial) or by being given a self-help book (UK trial); we did not consider these participants in this study. Further details regarding recruitment and eligibility can be obtained elsewhere [8,9], as well as in Table 1 and the flowchart in Figure 1. Table 2 shows the baseline characteristics of the participants at pretreatment for each clinical trial; the 2 trials differed on several baseline characteristics. Overall, participants in the UK trial were more likely to be single, be younger, report prior mental health problems, have previous experience of undergoing psychological treatment and using psychotropic medication, and have greater symptom severity on the FMPS CM, but not on the CPQ.

### Measures

The main outcome measure in both trials was the FMPS CM [5], comprising 9 items related to worries of making mistakes that are scored on a 5-point Likert scale (range 1-5). The FMPS CM has a Cronbach alpha of .88 [5]; in the 2 trials it was .85 (Swedish trial) and .74 (UK trial). Both trials also administered the CPQ [6], comprising 12 items associated with a specific construct of clinical perfectionism that are scored on a 4-point Likert scale (range 0-3). The CPQ has a Cronbach alpha of .73 [6]; in the 2 trials it was .66 (Swedish trial) and .74 (UK trial).

The secondary outcome measures differed between the 2 clinical trials but are nonetheless reported to examine the benefits on conditions other than perfectionism. In the Swedish trial, these were the 9-item Patient Health Questionnaire (PHQ-9) [13] and the 7-item Generalized Anxiety Disorder scale (GAD-7) [14]. The PHQ-9 has 9 items related to depression that are scored on a 4-point Likert scale (range 0-3), with a range in scores from 0 to 27. The PHQ-9 has a Cronbach alpha of .89 [13]; in Swedish trial it was .84. The GAD-7 has 7 items measuring worry and anxiety that are scored on a 4-point Likert scale (range 0-3), with a range in scores from 0 to 21. The GAD-7 has a Cronbach alpha of .92 [14]; in the Swedish study it was .87. The UK trial used the 21-item Depression Anxiety Stress Scale (DASS-21) [15]. The DASS-21 has 21 items measuring depression, anxiety, and stress that are scored on 4-point Likert scale (range 0-3), with a range in scores from 0 to 63. The DASS-21 has a Cronbach alpha of .88 [16]; in the UK study it was .91.

**Table 1:** Key characteristics of the clinical trials. FMPS CM: Frost Multidimensional Perfectionism Scale, Concern over Mistakes subscale; SMS: short message service text messaging.

Characteristic		Swedish trial	UK trial
Recruitment process		Self-referrals	Self-referrals
Cutoff criteria for maladaptive perfectionism		No	Yes (≥29 on the FMPS CM)
Telephone interview assessment		Yes (Mini-International Neuropsychiatric Interview)	No
**Inclusion criteria**			
	Minimum age	Yes (>18 years)	Yes (>18 years)
	Concurrent psychological treatment	Not allowed	Allowed
	Change in pharmacological treatment^a^	Not allowed	Allowed
	Other more severe conditions	Not allowed (eg, anorexia nervosa)	Allowed (except suicidality)
Randomization		Yes (once)	Yes (continuous)
Confirmation to commence treatment		Yes	No
Starting date		Simultaneous for all participants	Individual starting dates
Guidance from a therapist		Yes (on 2 predetermined weekdays)	Yes (weekly but with no specification)
Therapist level		Master’s degree	Mixed (undergraduate to PhD)
Therapist supervision		Yes (weekly)	Yes (monthly)
Feedback checked by supervisor		No	Yes
Treatment protocol		Egan et al [11]^b^	Egan et al [11]
Treatment period		8 weeks	12 weeks
Follow-up		12 months	6 months
Follow-up reminders		Telephone, email, and SMS	Email
Monetary compensation		No	Yes (£10)^c^
Ethics approval		Yes (Dnr 2015/419-31)	Yes (project identifier 6222:001)
Study protocol		No	Yes [12]
Registered at ClinicalTrials.gov		No	Yes (registration no. NCT02756871)
Informed consent obtained		Yes	Yes

^a^Any change 3 months prior to the screening process.

^b^Minor change in order of modules and greater emphasis on behavioral interventions.

^c^If participant completed posttreatment assessment.

**Table 2:** Baseline characteristics of the participants at pretreatment assessments. FMPS CM: Frost Multidimensional Perfectionism Scale, Concern over Mistakes subscale; N/A: not applicable.

Baseline characteristic		UK trial (n=62)	Swedish trial (n=78)	χ^2^	*df*	95% CI
Female sex, n (%)		49 (79.0)	64 (82.1)	0.2	1	N/A
Age (years), mean (SD)		28.6 (8.3)	34.22 (9.9)^a^	N/A	N/A	2.6 to -8.7
**Marital status, n (%)**				32.3	2	N/A
	Single	48 (77.4)^a^	23 (29.5)			
	Married/partner	14 (22.6)	52 (66.7)^a^			
	Divorced/widowed	0 (0.0)	3 (3.8)			
Prior mental health problem (yes), n (%)		20 (27.4)	4 (5.1)	17.9	1	N/A
Psychological treatment (yes), n (%)		16 (25.8)^a^	0 (0)	22.7	1	N/A
Psychotropic medication (yes), n (%)		11 (17.7)^a^	5 (6.4)	36.5	1	N/A
**Primary outcome measures, mean (SD)**						
	FMPS CM	36.7 (4.4)^a^	33.4 (6.4)	N/A	N/A	1.5 to 5.1
	Clinical Perfectionism Questionnaire	35.7 (4.7)	38.3 (4.6)^a^	N/A	N/A	–4.1 to –1.0

^a^The clinical trial with significantly higher values on a specific baseline characteristic.

**Table 3:** Modules and order of presentation in the clinical trials.

Module	Swedish trial	UK trial
1	Understanding your perfectionism	Understanding your perfectionism
2	Your own model, values, and motivation	Your own model, values, and motivation
3	Surveys and experiments	Surveys and experiments
4	Dealing with perfectionistic behaviors	New ways of thinking
5	New ways of thinking	Dealing with perfectionistic behaviors
6	Self-criticism and self-compassion	Self-criticism and self-compassion
7	Self-worth	Self-worth
8	Maintain and continue positive change	Maintain and continue positive change

The outcome measures were completed at pre- and posttreatment and follow-up (12 months for the Swedish trial and 6 months for the UK trial). The CPQ was also distributed weekly during the treatment period for the UK trial.

### Treatment and Therapists

In both clinical trials, the treatment content was derived from Egan et al [11], administered as an ICBT with guided self-help. It consisted of 8 modules, with 1 module delivered weekly, including psychoeducation, exercises, and homework assignments related to perfectionism. Each module was approximately 12 pages, totaling 121 pages. However, there were some minor differences between the 2 clinical trials in the order the modules were distributed (see Table 3). In addition, the Swedish trial put a greater emphasis on behavioral interventions than did the UK trial. Also, the treatment period was 8 weeks in the Swedish trial and 12 weeks in the UK trial. Therapists in the Swedish trial were Master’s degree students having completed 1.5 years of clinical training, compared with a mixed set of therapists in the UK trial (undergraduates, master’s degree students, doctoral candidates in clinical psychology, and PhDs). In both cases, the therapists provided the participants with weekly feedback, but while this was done on predetermined weekdays in the Swedish trial, this was not the case in the UK trial. The amount of therapist supervision also differed between the 2 clinical trials: weekly for the Swedish trial and monthly for the UK trial. Table 1 shows an overview of the differences. A study protocol for the UK trial is also available [12].

### Statistical Analysis

Given the significant baseline differences between the 2 trials, as well as the different lengths of follow-up, we did not combine the data for any analyses. We calculated a priori power for the 2 clinical trials to detect significant differences compared with the waitlist control at posttreatment, but not for a between-study difference at follow-up. We explored differences between the Swedish and the UK trial at baseline, as well as baseline predictors of completion of the self-report measures at follow-up (ie, dropouts, regardless of the number of modules they had completed during the treatment period), by using 2-sided independent *t* tests for continuous variables and chi-square tests for nominal variables.

We performed intention-to-treat analyses for the main and secondary outcome measures using multiple imputation to account for missing values and we conducted completer analyses using available data. The imputation models used all available self-report measures to create 10 imputed datasets. We then used analysis of covariance (ANCOVA) to investigate the change scores between pretreatment and follow-up, with scores at baseline as covariates, averaging the parameter estimates for the 10 analyses. All statistical analyses were done with IBM SPSS version 24.0.0.1 (IBM Corporation).

Using the results from the ANCOVAs, we calculated within-group effect sizes for the means of 2 assessments, divided by the pooled standard deviations. All results are presented with 95% CIs, where applicable, including effect sizes, and discussed in relation to similar findings in other clinical trials [17]. For the FMPS CM, we defined recovery as having a score at follow-up within 1 SD of that of the general population (<29 [18]; ie, a clinically significant change), with a change score also exceeding the RCI (ie, 1.96 times the standard error of the instrument [19]). This is assumed to reflect those participants moving from a dysfunctional to a functional distribution in terms of perfectionism. Furthermore, we determined improvement using only the RCI as a cutoff. Also, nonresponse corresponded to those participants not exceeding the RCI in any direction, while we calculated reliable deterioration using the RCI but in a negative direction [20].

All presented results concern the 6- and 12-month follow-ups. For a review of the outcomes at posttreatment, see Rozental et al [8] and Shafran et al [9].

## Results

### Attrition and Adherence

There was no statistical difference between the 2 clinical trials in attrition, defined as the number of participants who were randomly assigned but did not complete the assessment at follow-up. In the Swedish trial, 49 (63%) completed the follow-up self-report measures, compared with 35 (57%) in the UK trial (χ^2^_1_=0.6, *P*=.45). None of the baseline characteristics were related to attrition in either of the 2 clinical trials. With regard to adherence, defined as the number of completed modules during the treatment period, the 2 clinical trials differed significantly, with a mean difference of 3.14 modules (95% CI 2.31-3.97), demonstrating that the participants in the Swedish trial completed more modules than those in the UK trial, on average.

### Treatment Results

Table 4 shows the descriptive statistics for the primary and secondary outcome measures. The ANCOVAs for the intention-to-treat analyses revealed significant differences between pretreatment and follow-up for all of the primary and secondary outcome measures in both clinical trials. Table 5 shows within-group Cohen *d* effect sizes and their respective 95% CIs.

The completer analyses revealed significant differences for both clinical trials between pretreatment and follow-up, revealing a mean difference of 8.98 points (Swedish trial; 95% CI 7.07-10.89) and 10.35 points (UK trial; 95% CI 7.25-13.44) for the FMPS CM. Results were similar for the CPQ: 8.69 points (Swedish trial; 95% CI 6.61-10.77) and 11.10 (UK trial; 95% CI 9.14-13.07). For the secondary outcome measures, the mean differences in the Swedish trial were 3.57 points for the PHQ-9 (95% CI 2.28-4.86) and 3.22 points for the GAD-7 (95% CI 2.33-4.12). In the UK trial, the mean difference for the DASS was 8.78 points (95% CI 4.16-13.40).

**Table 4:** Observed and estimated scores for each primary outcome measure, by clinical trial, intention-to-treat analysis, and completer analysis. FMPS: Frost Multidimensional Perfectionism Scale, Concern over Mistakes subscale.

Measure and condition		Intention-to-treat analysis						Completer analysis					
		Pretreatment		Follow-up		Pretreatment		Follow-up	
		Mean (SD)	n	Mean (SD)	n	Mean (SD)	n	Mean (SD)	n
**Swedish trial^a^**													
	FMPS CM	33.42 (6.44)	78	25.14 (7.23)	78	33.42 (6.44)	78	23.61 (7.60)	49
	Clinical Perfectionism Questionnaire	38.26 (4.63)	78	29.63 (8.00)	78	38.26 (4.63)	78	29.51 (6.70)	49
	9-item Patient Health Questionnaire	9.59 (5.63)	78	6.45 (4.73)	78	9.59 (5.63)	78	5.47 (4.77)	49
	7-item Generalized Anxiety Disorder scale	7.83 (4.85)	78	4.95 (3.72)	78	7.83 (4.85)	78	3.84 (3.41)	49
**UK trial^b^**													
	FMPS CM	36.71 (4.42)	62	28.83 (7.80)	62	36.71 (4.42)	62	25.52 (8.07)	29
	Clinical Perfectionism Questionnaire	35.69 (4.73)	62	27.25 (6.44)	62	35.69 (4.73)	62	24.55 (5.25)	29
	21-item Depression Anxiety Stress Scale	26.31 (12.82)	62	19.89 (13.11)	62	26.31 (12.82)	62	15.93 (12.52)	27

^a^12-month follow-up.

^b^6-month follow-up.

**Table 5:** Within-group effect sizes, Cohen *d* (95% CI). FMPS: Frost Multidimensional Perfectionism Scale, Concern over Mistakes subscale.

Measure and condition		Intention-to-treat analysis	Completer analysis
		Cohen *d* (95% CI)	Cohen *d* (95% CI)
**Swedish trial**			
	FMPS CM	1.21 (0.86-1.54)	1.42 (1.01-1.81)
	Clinical Perfectionism Questionnaire	1.32 (0.97-1.66)	1.59 (1.17-1.98)
	9-item Patient Health Questionnaire	0.60 (0.28-0.92)	0.77 (0.40-1.14)
	7-item Generalized Anxiety Disorder scale	0.67 (0.34-0.99)	0.92 (0.54-1.29)
**UK trial**			
	FMPS CM	1.24 (0.85-1.62)	1.92 (1.38-2.43)
	Clinical Perfectionism Questionnaire	1.49 (1.09-1.88)	2.27 (1.70-2.80)
	21-item Depression Anxiety Stress Scale	0.50 (0.13-0.85)	0.82 (0.34-1.28)

### Improvement and Deterioration

Recovery on the FMPS CM was defined as those participants having a score at follow-up within 1 SD of the general population (<29; ie, clinically significant change) and exceeding the RCI. According to this definition, 29/49 (59%) participants in the Swedish trial and 15/35 (43%) in the UK trial met the criteria for recovery at follow-up. Improvement—that is, having a change score beyond the RCI at follow-up—was achieved by 31/49 (63%) in the Swedish trial and 18/35 (51%) in the UK trial. Meanwhile, 17/49 (35%) in the Swedish trial and 11/35 (31%) in the UK trial did not respond. In none of these cases was there a significant difference between the 2 clinical trials: χ^2^_2_ range 3.09 to 3.99, *P* value range .14 to .21. Only 1/84 (1%) participant deteriorated.

## Discussion

### Principal Findings

This study evaluated the long-term benefits of ICBT with guided self-help for perfectionism, indicating that the results at follow-up were similar to and possibly even somewhat improved from posttreatment. This suggests that ICBT with guidance from a therapist could help individuals manage and overcome their perfectionism in the long term. Compared with the findings of Lloyd et al [4], in this study the within-group effect sizes for perfectionism are similar, although that systematic review and meta-analysis included data only from pre- and posttreatment and not from follow-up. This is also true for depression and anxiety, with average Hedges *g* effect sizes being 0.64 (95% CI 0.35-0.92) and 0.52 (95% CI 0.23-0.81), respectively. In other words, the findings from our study are in line with previous research, suggesting that some long-term benefits can be achieved for anxiety, depression, and stress as well. Given that we could not combine the outcomes from the 2 clinical trials due to the differences in several key characteristics, most notably not implementing the same follow-up period, the result s should be interpreted with caution. Nevertheless, the results from this study are comparable with those of Egan et al [7] at 6-month follow-up, although, in that study, unguided self-help yielded much lower effects than face-to-face CBT. Therefore, the use of guided self-help when providing ICBT for perfectionism might be assumed to be better than unguided self-help for outcome. This idea was supported by a study on the differences between various levels of guidance by a therapist in ICBT for patients with depression [21] and, similarly, by a systematic review and meta-analysis of self-guided interventions for obsessive-compulsive disorder [22], indicating that more support yields larger effects overall. However, Titov et al [23] did not find such a difference in outcome, arguing that carefully controlled ICBT without any support can be just as beneficial. More research on the importance of guidance in relation to the treatment of perfectionism with ICBT is thus warranted, including an investigation of its influence on adherence.

The rates of recovery at follow-up, that is, those participants meeting the criteria of moving from a dysfunctional to a functional distribution in terms of perfectionism (clinically significant change and exceeding the RCI) were 59% (FMPS CM) in the Swedish trial and 43% (FMPS CM) in the UK trial. This can be compared with the results of Egan et al [7] of 67% for face-to-face CBT and 40% for unguided self-help. However, given that, to our knowledge, no other clinical trial has investigated recovery at follow-up, these numbers should be interpreted cautiously and warrant replication. Furthermore, given the high rate of attrition in this study, the estimates of recovery might be unreliable because we derived them from those participants completing the follow-up assessment, possibly inflating the actual rates. In addition, it is unclear whether the cutoff for determining clinically significant change (<29 on the FMPS CM [18]) is sensitive enough to accurately differentiate those belonging to a clinical population from those belonging to a nonclinical population. In this sense, recovery in this study should primarily be regarded as reaching a statistical criterion, in line with the recommendations by Jacobson and Truax [24], rather than true recovery in terms of no longer fulfilling the criteria for a psychiatric disorder. Whether participants are in fact recovered from perfectionism is, however, an issue that requires both more empirical data and a better conceptual idea of where the dysfunctional and functional distributions meet. Nevertheless, the recovery rates indicate that ICBT works well and does have an impact on perfectionism that should be clinically relevant.

### Study Limitations

This study makes an important contribution to the research on the treatment of perfectionism, as it is one of few studies that included follow-up data and the only one to date, to our knowledge, with follow-up at 12 months. There are, however, also limitations that need to be addressed. First, issues related to the design limit the conclusions that can be made. The 2 clinical trials differed on some key characteristics, including different length of the treatment period (8 vs 12 weeks), therapist experience, therapist supervision, and, especially, the timing of the follow-up assessment. This was primarily due to different conventions and logistical issues among the researchers involved, meaning that the samples could not be combined and thus limit power. Nevertheless, given the many similarities between them in terms of treatment content and delivery, the findings are arguably relevant to present together. Additionally, there was no comparison group. However, replicating this study is warranted, possibly by recruiting a larger and more heterogeneous sample in the context of a randomized controlled trial.

Second, attrition was high in both clinical trials, with only 63% (Swedish trial) and 57% (UK trial) completing the self-report measures at 6- and 12-month follow-up, respectively, potentially affecting the conclusions that can be drawn. This can be compared with a study on ICBT for procrastination [25], which had 32% attrition at 12-month follow-up, suggesting that our clinical trials both had higher rates of dropouts. In addition, an individual patient data meta-analysis of 10 studies on ICBT for depression indicated that 40% dropped out before completing one-fourth of the modules [26]. Multiple imputation was used to account for missing values, given the indication that data were missing at random. In our study, none of the baseline characteristics were associated with attrition, but this does not preclude other variables that we did not explore from being related to completing the self-report measures at follow-up. Preventing attrition is thus important, and future research should try to implement ways of improving these rates, perhaps by adding more support or the use of tailored modules [27]. Additional issues related to the different attrition rates in the 2 clinical trials have also been addressed by Shafran et al [9], such as not explicitly asking the participants to confirm their participation and the absence of a telephone interview assessment in the UK trial, aspects that may have to be addressed to a greater extent in future studies.

Third, perfectionism is not a psychiatric disorder in itself [1]. This complicates the issue of assessing eligibility to participate in a clinical trial, but also of determining whether it is a condition that actually requires a stand-alone treatment like the one provided. However, given the close connection with eating disorders, depression, and anxiety disorders [3], it is reasonable to assume that elevated perfectionism is a common denominator for many psychiatric disorders. Hence, interventions targeting its mechanisms should be beneficial for many individuals by providing a more transdiagnostic approach. This is supported by the finding that benefits can be observed on many different outcomes in clinical trials of perfectionism, such as depression [7-9]. Still, more research needs to be done to investigate whether a transdiagnostic approach adds something to a disorder-specific treatment, for instance comparing their efficacy in a head-to-head comparison for a particular psychiatric disorder.

Fourth, the participants in this study were all self-referred, and although they scored high on perfectionism at pretreatment, they may not be representative of individuals with this problem in general. Arnberg et al [28] stressed that most individuals receiving help via ICBT have a high educational level and are more likely to be women and of a particular socioeconomic group, possibly limiting the generalizability of the results. However, Titov et al [29] compared the baseline characteristics of individuals receiving ICBT with both those receiving CBT at an outpatient clinic and those taking an epidemiological survey, noting that the differences were small and not necessarily important. In comparison with the general population, those receiving treatment, regardless of format, had higher severity levels overall, but ICBT and CBT did not differ from each other. Also, with the only exceptions of age, sex, and marital status, such aspects as educational level and employment status were not different between ICBT and CBT treatment groups, suggesting that there may not be anything specific to receiving treatment via the internet with regard to those who seek help in general. Further research should nonetheless be performed on recruitment and diversity, preferably by limiting the number of inclusion and exclusion criteria and by reaching out to a more heterogeneous sample.

### Conclusion

We examined the long-term benefits of ICBT with guided self-help for perfectionism, depression, and anxiety. The results at follow-up were comparable with posttreatment assessment, obtaining medium to large within-group effect sizes. The results from these 2 different cases of ICBT with guided self-help are thus promising but warrant replication using a larger and more heterogeneous sample.

## Abbreviations

ANCOVA: analysis of covariance
CBT: cognitive behavioral therapy
CPQ: Clinical Perfectionism Questionnaire
DASS-21: 21-item Depression Anxiety Stress Scale
FMPS CM: Frost Multidimensional Perfectionism Scale, Concern over Mistakes subscale
ICBT: internet-based cognitive behavioral therapy
PHQ-9: 9-item Patient Health Questionnaire
RCI: Reliable Change Index
